# Rickettsial fever leading to upper disphagya: A rare neurovascular conflict between vertebral artery and glossopharyngeal nerve

**DOI:** 10.1016/j.idcr.2021.e01250

**Published:** 2021-08-09

**Authors:** Jorge Lourenco, Joana Coelho, Pedro Ribeiro

**Affiliations:** Internal Medicine Department, Centro Hospitalar e Universitario de Coimbra, Portugal

**Keywords:** Rickettsia, Neuropathy, Dysphagia, Fever

## Abstract

Rickettsial fevers are a common type of zoonosis with potential neurological involvement, mostly in form of meningoencephalitis. However, there are some rare cases that could compromise cranial nerves function through vascular inflammation. In this paper, we describe a rare case of dysphagia related to glossopharyngeal involvement by juxtaposed vertebral artery vasculitis.

## Introduction

Rickettsial infections are an important and often underdiagnosed cause of undifferentiated febrile illness. These are obligate intracellular gram-negative bacteria, distributed throughout the world and divided into four phylogenetic groups, namely ancestral (*R.bellii, R.canadensis*), typhus (*R.prowazekii, R.typhi*), transitional (*R.akari, R.felis*), and spotted fever groups (*R.rickettsii, R.conorii, R.sibirica*). The major route of transmission typically involves a wide range of arthropod vectors such as fleas, ticks and mites [1].

Indirect fluorescent-antibody is currently the diagnostic method of choice, as well as eschar swab and biopsy; real-time PCR assays could be an alternative, particularly in early stages of the disease. The infection-associated symptoms are non-specific and vary in severity from mild or self-limiting (fever, headache, myalgia, arthralgia, and malaise) to fulminant life-threatening conditions such as splenic rupture or central nervous system involvement [1,2]. Occasionally characteristic skin rash is found on physical examination. Several authors have described neurological sequelae in patients who survived from complicated forms of this infection, such as septic shock and multiple organ dysfunction syndrome. Those patients can develop hemiplegia, deafness, visual disturbance, slurred speech, mental confusion, and cranial neuropathies that may persist for a few weeks following recovery [1,2]. In this article we present a rare case of a vasculitic process involving vertebral artery leading to glossopharyngeal compression.

## Case report

A 61-year-old Ukrainian male living in Portugal was admitted in Intensive Care Unit of our hospital due to an episode of septic shock related to a R. rickettsi infection confirmed with ELISA serology test (IgM antibody index = 19.2; >11 is positive) and IgG (antibody index = 10.4; equivocal), associated with haematological and hepatic disfunction.

He was treated with doxycycline 100 mg bid and after 5 days of admission, he was transferred to Internal Medicine Department for further evaluation. At that time, the patient showed difficulty to swallow food or beverages consisting of upper dysphagia and referred a stabbing pain at the base of the tongue. He denied abdominal pain, vomiting and showed no diarrhoea nor constipation.

**Fig. 1:**
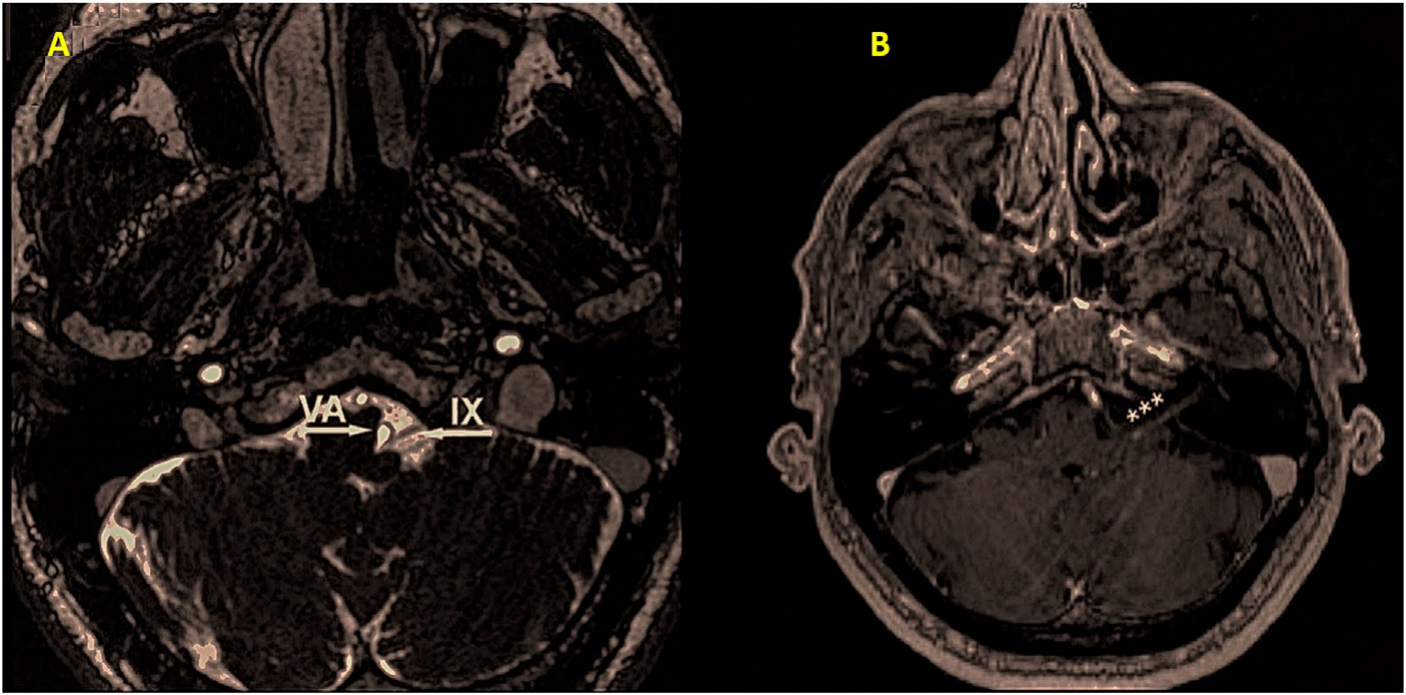
(A) T2-weighted and (B) T1-weighted contrast-enhanced images of left glossopharyngeal (IX) nerve after gadolinium, confirming neurovascular conflict with the vertebral artery (VA), which seems to compress the proximal portion of the nerve.

His neurologic examination confirmed patient’s complaints, with no additional deficits identified. Given the persistence of symptoms, a nasogastric tube was required for correct feeding and hydration. His blood tests included normal ionogram as well as normal levels of B12 vitamin and folic acid. He underwent brain CT and upper endoscopy, which showed no significant findings. However, the patient’s brain MRI confirmed juxtaposition of the left glossopharyngeal nerve and the vertebral artery, with abnormal glossopharyngeal nerve contrast enhancement (Fig. 1). Neurologist evaluation concluded that this could be considered a post-infectious vasculitic process and began treatment for glossopharyngeal neuralgia with oxcarbazepine 300 mg three times daily. The patient showed pain relief after the first week of treatment and recovered completely of dysphagia during 3 weeks of rehabilitation workup.

## Discussion

Clinical manifestations of rickettsioses often relate to vasculitis, despite direct invasion of CNS has been described in patients with acute neurological complications [2]. Despite being an acute undifferenciated febrile illness, they typically do not present laboratory or imaging features of meningeal inflammation just as described in our case report, where symptoms related to cranial neuropathies could develop through immune-mediated mechanisms caused by widespread endothelial inflammation and progression of vasculitic process [2,3]. Those manifestations are quite rare in rickettsioses with only a few cases published, namely ocular [4,5], facial [6] and vestibulocochlear nerve palsies [6,7]. Prompt treatment initiation is key to improve clinical outcome. Use of corticosteroids is expected to enhance control of local inflammation, but it does not exclude the use of antibiotics [3]. Doxycycline is preferred over other tetracyclines and should be administered 3–5 days after defervescence. This is to our knowledge the first case published of secondary glossopharyngeal involvement in a case of Rickettsia infection.

## Declaration of Competing Interest

The authors report no declarations of interest.

## Sources of funding for your research

There are no funding sources or sponsors related to the present work.

## Patient’s consent

Written informed consent was obtained from the patient for publication of this case report and accompanying images. A copy of the written consent is available for review by the Editorin-Chief of this journal on request.

## Authors contribution

All authors had a role in writing and revising this manuscript
